# Individual and community-level risk factors for HIV stigma in 21 Zambian and South African communities: analysis of data from the HPTN071 (PopART) study

**DOI:** 10.1097/QAD.0000000000001757

**Published:** 2018-03-21

**Authors:** James R. Hargreaves, Shari Krishnaratne, Hlengani Mathema, Pamela S. Lilleston, Kirsty Sievwright, Nomtha Mandla, Tila Mainga, Redwaan Vermaak, Estelle Piwowar-Manning, Ab Schaap, Deborah Donnell, Helen Ayles, Richard J. Hayes, Graeme Hoddinott, Virginia Bond, Anne Stangl

**Affiliations:** a London School of Hygiene and Tropical Medicine, London, UK; b Desmond Tutu TB Centre, Department of Paediatrics and Child Health, Faculty of Medicine and Health Sciences, Stellenbosch University, Cape Town, South Africa; c International Centre for Research on Women, Washington DC; d Department of Pathology, Johns Hopkins University School of Medicine, Baltimore, Maryland, USA; e Zambart, The School of Medicine, University of Zambia; f Statistical Centre for HIV/AIDS Research and Prevention (SCHARP), Fred Hutchinson Cancer Research Center, Seattle, Washington, USA.

**Keywords:** Africa, AIDS, epidemiology, HIV, implementation science, stigma

## Abstract

**Objective:**

To describe the prevalence and determinants of HIV stigma in 21 communities in Zambia and South Africa.

**Design:**

Analysis of baseline data from the HPTN 071 (PopART) cluster-randomized trial. HIV stigma data came from a random sample of 3859 people living with HIV. Community-level exposures reflecting HIV fears and judgements and perceptions of HIV stigma came from a random sample of community members not living with HIV (*n* = 5088), and from health workers (HW) (*n* = 851).

**Methods:**

We calculated the prevalence of internalized stigma, and stigma experienced in the community or in a healthcare setting in the past year. We conducted risk-factor analyses using logistic regression, adjusting for clustering.

**Results:**

Internalized stigma (868/3859, prevalence 22.5%) was not associated with sociodemographic characteristics but was less common among those with a longer period since diagnosis (*P* = 0.043). Stigma experienced in the community (853/3859, 22.1%) was more common among women (*P* = 0.016), older (*P* = 0.011) and unmarried (*P* = 0.009) individuals, those who had disclosed to others (*P* < 0.001), and those with more lifetime sexual partners (*P* < 0.001). Stigma experienced in a healthcare setting (280/3859, 7.3%) was more common among women (*P* = 0.019) and those reporting more lifetime sexual partners (*P* = 0.001) and higher wealth (*P* = 0.003). Experienced stigma was more common in clusters wherever community members perceived higher levels of stigma, but was not associated with the beliefs of community members or HW.

**Conclusion:**

HIV stigma remains unacceptably high in South Africa and Zambia and may act as barrier to HIV prevention and treatment. Further research is needed to understand its determinants.

## Abstract 2

Supplemental Digital Content is available in the text

## Introduction

HIV stigma is present whenever HIV infection is linked to negative stereotypes that mark a person living with HIV as different from rest of the population; a separation of ‘them’ from ‘us.’ This separation then leads to status loss, which can result in negative outcomes for people living with HIV (PLHIV) [1]. Stigma experienced by PLHIV can include being gossiped about, insulted or physically assaulted in communities and healthcare settings [2]. Internalized stigma occurs whenever PLHIV apply the same negative feelings to themselves and can have mental health consequences [3–6]. HIV stigma infringes human rights and can inhibit access to HIV testing and care [7,8].

Few studies have compared data both from those whose beliefs and behaviours are thought to drive the stigmatization process and also from those who experience it. The community-level factors that give rise to stigma are under-studied [9,10]. Stigma theories suggest that the beliefs and behaviours of community members and health workers (HW) are drivers of stigma [11,12], but there are few quantitative data to support this.

We analysed baseline data from a large cohort study of HIV stigma nested within the HPTN 071 (PopART) trial [13,14]. The outcomes of interest were experienced and internalized stigma reported by PLHIV. We first explored individual-level risk factors. We then investigated the hypothesis that stigma reported by PLHIV was more common in communities with higher levels of fear and judgement towards PLHIV. Finally, we investigated whether stigma reported by PLHIV was more common in communities with more perceived stigma reported by community members and HW.

## Methods

Twenty-one urban communities (9 in South Africa, 12 in Zambia) were purposively selected to take part in the HPTN 071 (PopART) cluster-randomized trial. The trial tests the impact of a combination HIV prevention package, including universal door-to-door HIV testing and offer of antiretroviral therapy (ART) regardless of CD4^+^ cell count, on HIV incidence. Using a ‘parallel’ approach [15,16], we combined outcome and exposure data from three separate populations who were interviewed in two data collection activities (see Appendix S1 and the following for further details).

### Stigma outcome measurement

Outcome data came from individuals recruited to the HPTN 071 (PopART) Population Cohort who both self-reported living with HIV and were laboratory-confirmed as HIV-positive. We refer to this group as PLHIV. HIV status was determined by testing blood samples drawn from consenting survey participants. Blood samples were analysed in-country using a single fourth generation assay (Architect HIV Ag/Ab Combo Assay, Abbott Diagnostics, Delkenheim Germany). Further testing was performed at the HPTN Laboratory Center (Baltimore, Maryland, USA). Samples that had reactive results in-country were tested with a second fourth generation assay (GS HIV Combo Assay, Bio-Rad Laboratories, Redmond, Washington, USA). Samples with discrepant/discordant test results were tested with additional assays to determine HIV status. The cohort was enrolled between November 2013 and March 2015. In each community, household listing generated a sample frame [13]. The target sample size was 2500 individuals per community, of whom 15% were expected to be living with HIV. In randomly sampled households, one adult resident aged 18–44 years was selected at random. Participants completed an interviewer-administered questionnaire with data captured on an electronic device. Participants were asked if they had an HIV-test previously, and if comfortable to do so, to share the result of their last test [13]. Participants were also offered voluntary counselling and testing using rapid HIV-test kits. Individuals testing positive were referred to a government health facility.

PLHIV were asked about their experiences of stigma. Item and response wording are held in Table S1 (Appendix S1). Item wording was informed by previous harmonization on measures of HIV stigma [12]. PLHIV responded to three items on internalized stigma (see Table 2 for item and response wording). Responses were summarized into a binary variable describing whether participants agreed to feeling any of three manifestations of internalized stigma. Five items captured experienced stigma in a community setting, and three captured stigma experienced in healthcare settings. Precoded response categories identified the frequency of experiences during the last year. These items were collapsed to create two binary variables capturing experience of stigma, in the community or in a healthcare setting, during the last year. Three thousand eight hundred and fifty-nine PLHIV had complete data on all 11 stigma items and on all sociodemographic variables (Figure S1a).

### Exposure measurement

Individual-level exposure data came from the same interviews with PLHIV as the outcome data. Exposures considered included sex, age, education, marital status, HIV treatment (i.e. ever started ART), HIV-status disclosure, sexual behaviour and household wealth.

We also measured community-level characteristics reflecting the level of HIV fear and judgement and the perceptions of stigma reported by community members and HW. The HIV fear and judgement items captured participants’ attitudes towards PLHIV. The perceptions of stigma questions reflected whether participants perceived that stigma was occurring rather than reflecting their attitudes. Data were collected from a random 20% sample of the Population Cohort described above. We included data from participants who did not have a confirmed HIV-positive blood test or self-report being HIV-positive (5088 individuals, range 161–441 per community). We refer to this group as community members (CM). We asked CM about their fears and judgement toward PLHIV (three items), levels of perceived stigma in communities (five items), and levels of perceived stigma in healthcare settings (two items) (Table S1 and Figure S1b). Each question was asked on a 4-point Likert scale scored as follows (strongly disagree 0, disagree 1, agree 2, strongly agree 3). Three scores were calculated for each individual as the mean of the item responses. Each score could theoretically range from 0 to 3, with 0 representing all items being responded to as ‘Strongly Disagree’ and 3 representing all ‘Strongly Agree’. Cluster summary variables were calculated as the mean of the individual responses, with higher scores representing communities with a greater presence of stigmatizing attitudes or a higher level of perceived stigma. Thus, for any community, a score of ‘1’ would mean that the average response to all items across all individuals was to ‘Disagree’ with the statements.

Data on the beliefs and perceptions of HW came from the baseline survey for a separate cohort study conducted as part of the trial [15]. We recruited consenting health-facility staff and community HW delivering HIV-related services. We also collected data from new trial intervention staff (known as ‘CHIPS’ [13]) but excluded these from this analysis as these individuals had only just begun to work in the communities at the time of data collection. We included data only from HW who did not self-report being HIV-positive. We refer to this group as HW. Again, three scores were developed reflecting HIV fear and judgement (five items), perceptions of the stigmatizing behaviours of their co-workers (four items) and perceptions of stigma in the community (five items). Scoring at individual and community levels was as above. Some 851 HW contributed data to this analysis (range 13–77 per community; (Table S1 and Figure S1c).

### Statistical analysis

We summarized PLHIV characteristics in each country and describe variation in stigma prevalence by cluster (range). There was one cluster with a low sample size (*n* = 5 PLHIV) leading to outlier values. Wherever relevant we present the outlier value separately, and the cluster range excluding this value. We calculated Cronbach's alpha to assess inter-item agreement.

In risk-factor analysis, we assessed whether both individual-level and cluster-level characteristics were associated with each of the three PLHIV stigma outcomes (internalized, experienced in the community, experienced in a healthcare setting). We used logistic regression and report the odds ratios and 95% confidence intervals and Wald-test values for each risk factor for each of the three outcomes in turn. Regression analyses were carried out excluding categories wherever a response was ‘Don’t know’ (ever started ART) or missing (time since first positive HIV test, first time had sex, number of sexual partners and condom use). We examined the impact of missing data on these four risk factors on the three stigma outcomes and found that PLHIV with missing data were less likely to report HIV stigma. We adjusted the standard errors using the vce (cluster) command in Stata v14 (StataCorp. College Station, Texas, USA) to reflect the study design, and adjusted all analyses for sex and age. In Appendix S2, we report on a sensitivity analysis restricted to 2342 PLHIV who, at the time of recruitment to the study, had not yet had a visit from the trial intervention team. We were concerned that this visit may influence stigma reporting. In summary, the prevalence of stigma was largely unchanged and although there were changes in point estimates and significance values for individual variables, there were no systematic differences of interpretation.

For individual-level risk factor analysis, we included sociodemographic and sexual behaviour characteristics that have been associated with stigma in previous analyses [17,18]. For cluster-level risk factor analysis we hypothesized that PLHIV-reported levels of internalized stigma and stigma experienced in the community would be correlated with the level of HIV fear and judgement reported by CM, and with perceived levels of community stigma reported by CM and HW. We hypothesized that stigma reported by PLHIV in healthcare settings would be correlated with the level of HIV fear and judgement reported by HW, with perceived levels of stigma in healthcare settings reported by CM, and with perceptions of stigma among co-workers reported by HW. To aid interpretation, we produced cluster-level scatter plots of the associations between the prevalence of each type of stigma and the cluster-level exposures. Each cluster was represented by a circle proportional in size to the number of PLHIV included in the analysis. We added fit lines from unadjusted, cluster-level linear regressions of the associations weighted by the size of the PLHIV population in each cluster.

### Ethics

The HPTN 071 (PopART) trial [Division of AIDS (DAIDS) #11865 and Clinical Trials registration number NCT01900977] and the stigma ancillary study (DAIDS # HPTN 071a) received institutional review board (IRB) approval from the London School of Hygiene and Tropical Medicine LSHTM, the Health Research Ethics Committee, Stellenbosch University, and the Biomedical Research Ethics Committee at the University of Zambia. Written informed consent was sought and obtained from all participants for all aspects of the research.

## Results

### Sociodemographic and behavioural characteristics of people living with HIV

Outcome data were available from 3859 PLHIV (ranging from 60 to 411 PLHIV by study community, with one outlier community, which only had five PLHIV). Most participants were women (86% from Zambia, 90.9% from South Africa; Table 1). Most had attended secondary school, with more doing so in South Africa than Zambia (80.6 versus 45.8%). More Zambian participants were married (61.7%) than in South Africa (30.8%). Approximately 70% of PLHIV reported that they had ever started antiretroviral therapy in both countries. Less than 10% of individuals reported that they had not disclosed their HIV status to anyone. Among those who had, disclosure was most commonly to a family member or a marital or sexual partner. Some 37.6% of PLHIV in South Africa, and 41.9% in Zambia, had been diagnosed 1–5 years previously. Characteristics of sexual behaviour most commonly reported were age at first sex 16–18 years, two to five lifetime sexual partners and condom use at last sex.

**Table 1:** Sociodemographic and behavioural characteristics of 3859 people living with HIV who responded to survey items measuring internalized and experienced stigma.

	South Africa,	Zambia,
Individual characteristics	*n*/1704 (%)	*n*/2155 (%)
Sex		
Male	155 (9.1%)	301 (14.0%)
Female	1549 (90.9%)	1854 (86.0%)
Age (years)		
24 or less	166 (9.7%)	257 (11.9%)
25–34	827 (48.5%)	950 (44.1%)
35–44	711 (41.7%)	948 (44.0%)
Education		
Did not complete secondary	292 (17.1%)	1052 (48.8%)
Completed secondary	1374 (80.6%)	986 (45.8%)
Further	38 (2.2%)	117 (5.4%)
Marital status		
Not married	1180 (69.2%)	825 (38.3%)
Married	524 (30.8%)	1330 (61.7%)
Ever started ART		
Yes	1185 (69.5%)	1505 (69.8%)
No	172 (10.1%)	168 (7.8%)
Do not know	347 (20.4%)	482 (22.4%)
Disclosed to^a^		
No-one	118 (6.9%)	182 (8.4%)
Husband/wife/sexual partner	644 (37.8%)	1062 (49.3%)
Family member	1313 (77.1%)	1530 (71.0%)
Friend/neighbour/colleague	301 (17.7%)	221 (10.3%)
Religious leader/worker	26 (1.5%)	64 (3.0%)
Healthcare worker	83 (4.9%)	106 (4.9%)
Other	11 (0.6%)	25 (1.2%)
How old were you the first time you had sex		
11–15	230 (13.5%)	402 (18.7%)
16–18	891 (52.3%)	963 (44.7%)
19–24	406 (23.8%)	558 (25.9%)
25+	12 (0.7%)	38 (1.8%)
Skipped/missing	165 (9.7%)	194 (9.0%)
How long has it been since your first positive HIV test?		
0–11 months	255 (15.0%)	495 (23.0%)
1–5 years	641 (37.6%)	902 (41.9%)
More than 5 years	369 (21.7%)	385 (17.9%)
Skipped/missing	439 (25.8%)	373 (17.3%)
How many sexual partners have you had in your lifetime?		
1	224 (13.1%)	415 (19.3%)
2–5	874 (51.3%)	1282 (59.5%)
6–10	242 (14.2%)	168 (7.8%)
11–15	29 (1.7%)	23 (1.1%)
16–20	17 (1.0%)	11 (0.5%)
More than 20	12 (0.7%)	28 (1.3%)
Skipped/missing	306 (18.0%)	228 (10.6%)
The last time you had sex, did you use a condom		
No	274 (16.1%)	727 (33.7%)
Yes	1039 (61.0%)	818 (38.0%)
Skipped/missing	391 (22.9%)	610 (28.3%)
Wealth tertile		
Lowest	772 (45.3%)	775 (36.0%)
Middle	614 (36.0%)	969 (45.0%)
Highest	318 (18.7%)	411 (19.1%)
Visit from community HIV care providers (CHiPs)		
No	1237 (72.6%)	1105 (51.3%)
Yes	369 (21.7%)	996 (46.2%)
Missing	98 (5.8%)	54 (2.5%)

^a^Multiple responses could be given and totals do not add up to 100. Responses were aggregated as a binary variable for subsequent analysis to reflect whether individuals reported that they had disclosed their HIV status to no-one or to anyone.

### Prevalence of stigma reported by people living with HIV

Twenty-two and a half percent of PLHIV (868/3859) agreed or strongly agreed with one of the three items reflecting internalized stigma (cluster range 1.9–35.4%, outlier cluster 80%, Table 2). Agree responses were more common than strongly agree. Inter-item agreement was high (Cronbach's alpha 0.82). Internalized stigma was more common in Zambian than South African clusters (25.9 versus 18.2%, *P* < 0.001). 22.1% of PLHIV (853/3859) reported at least one of the five items reflecting stigma experienced in the community (cluster range 6.4–36.8%, outlier cluster 80%). Across items, 6–9.2% of individuals reported the experiences had not happened because their status was unknown. Most events were experienced once or a few times rather than often. Inter-item agreement was again high (alpha 0.92). Reported experiences of stigma in the community were more common among Zambian than South African clusters (24.7 versus 18.8%, *P* < 0.001). 7.3% of PLHIV (280/3859) reported at least one of the three items reflecting healthcare setting experiences of stigma in healthcare settings (1–21.8%, outlier cluster 60%), and more commonly in South African than Zambian communities (8.7 versus 6.1%, *P* < 0.001). Inter-item agreement was again high (alpha 0.90). Overall, the prevalence of reporting any type of stigma was 35.5% (1371/3859).

**Table 2:** Responses from people living with HIV to items on internalized and experienced stigma items (*n* = 3859).

Internalized stigma	Strongly disagree	Disagree	Agree	Strongly agree
I have lost respect or standing in the community because of my HIV status	1296 (33.6%)	2093 (54.2%)	316 (8.2%)	154 (4.0%)
I think less of myself because of my HIV status	1224 (31.7%)	2138 (55.4%)	340 (8.8%)	157 (4.1%)
I have felt ashamed because of my HIV status	1295 (33.6%)	2046 (53.0%)	364 (9.4%)	154 (4.0%)
‘Current internalized stigma’: Responding agree or strongly agree to any of the above	868/3859 (22.5%); South Africa (18.2%) versus Zambia (25.9%), *P* < 0.001; Cronbach's alpha (0.82); cluster range (1.9%–35.4%); outlier 80.0%			

Frequency of experienced stigma (any setting)	Never	Not disclosed	Once	A few times	Often
People have talked badly about me because of my HIV status	2908 (75.4%)	356 (9.2%)	233 (6.0%)	272 (7.0%)	90 (2.3%)
Someone else disclosed my HIV status without my permission	3119 (80.8%)	258 (6.7%)	277 (7.2%)	163 (4.2%)	42 (1.1%)
I have been verbally insulted, harassed and/or threatened because of my HIV status	3304 (85.6%)	234 (6.1%)	131 (3.4%)	156 (4.0%)	34 (0.9%)
I have been physically assaulted because of my HIV status	3455 (89.5%)	230 (6.0%)	66 (1.7%)	86 (2.2%)	22 (0.6%)
I have felt that people have not wanted to sit next to me, for example, on public transport, at church or in a waiting room because of my HIV status	3387 (87.8%)	330 (8.6%)	76 (2.0%)	49 (1.3%)	17 (0.4%)
‘Experienced any stigma in past year’: responding once, a few times or often (‘ever’) to any of the above	853/3859 (22.1%); South Africa (18.8%) versus Zambia (24.7%), *P* < 0.001; Cronbach's alpha (0.92); cluster range (6.4–36.8%); outlier 80.0%				

Frequency of experienced stigma (health setting)	Never	Not disclosed	Once	A few times	Often
I have been denied health services because of my HIV status	3627 (94.0%)	114 (3.0%)	55 (1.4%)	51 (1.3%)	12 (0.3%)
Healthcare workers talked badly about me because of my HIV status	3558 (92.2%)	121 (3.1%)	101 (2.6%)	66 (1.7%)	13 (0.3%)
A health worker disclosed my HIV status without my permission	3593 (93.1%)	103 (2.7%)	90 (2.3%)	64 (1.7%)	9 (0.2%)
‘Experienced healthcare setting stigma in last year’: responding once, a few times or often (‘ever’) to any of the above	280/3859 (7.3%); South Africa (8.7%) versus Zambia (6.1%), *P* = 0.002; Cronbach's alpha (0.90); cluster range (1.0–21.8%); outlier 60.0%				
‘Current internalized stigma, experienced any stigma in past year or healthcare setting stigma in last year’	1371/3859 (35.5%); cluster range (11.4–55.8%); outlier 100.0%				

### Community-level characteristics

The cluster-score reflecting fear and judgement towards PLHIV reported by CM was 0.9 in South Africa and 0.8 in Zambia (Table 3), with substantial variation between clusters (range 0.4–1.2). Note that a score of 0.9 represents that across all communities the average participant response was closer to ‘Disagree’ (1) than to ‘Strongly Disagree’ (0). On average, CM also ‘disagreed’ with statements regarding the perception that stigma was present in communities (1.2 South Africa, 1.3 Zambia) and healthcare settings (1.1 South Africa, 0.9 Zambia), again with large intercluster variation. HW on average disagreed with statements reflecting HIV fear and judgement (mean 0.8 in both South Africa and Zambia), and there was less variation across clusters (range 0.6–1.1). HW reflected a somewhat higher score with regard to statements about the perception of stigma in communities (mean 1.5 South Africa, 1.4 Zambia), but disagreed on average with statements about their co-workers stigmatizing PLHIV (0.8 South Africa, 1.0 Zambia), with moderate variation across clusters. For all scores, consistency among items was moderate to high (Cronbach's alpha 0.66–0.84, Table 3).

**Table 3:** Cluster level characteristics describing the beliefs and perceptions of community members and health workers.

Scores	*N*/population (range)	Mean score – South Africa (cluster range)	Mean score – Zambia (cluster range)	Cronbach's alpha (items)
		9 clusters	12 clusters	
Average level of fear and judgement reported by CM	5088 (127–382)	0.9 (0.4–1.2)	0.8 (0.4–1.1)	0.74 (3)
Average level of perceived HIV stigma in the community reported by CM	5088 (127–382)	1.2 (0.6–1.9)	1.3 (0.8–1.6)	0.84 (5)
Average level of perceived HIV stigma in healthcare settings reported by CM	5088 (127–382)	1.1 (0.6–1.8)	0.9 (0.6–1.3)	0.76 (2)
Average level of HIV fear and judgement reported by HW	851 (13–77)	0.8 (0.6–1.1)	0.8 (0.6–0.9)	0.67 (5)
Average level of perceived HIV stigma in the community reported by HW	851 (13–77)	1.5 (1.3–1.8)	1.4 (1.3–1.6)	0.66 (5)
Average level of perceived co-workers stigmatizing behaviour reported by HW	851 (13–77)	0.8 (0.6–1.1)	1.0 (0.8–1.1)	0.76 (4)

CM, community members not living with HIV; HW, health workers not living with HIV. All scores have a theoretical range from 0 (all answers of all individuals ‘Strongly Disagree’) to 3 (all answers of all individuals ‘Strongly Agree.’ A mean score of 1 indicates a person that, on average, responds ‘Disagree’ to items within a score; a mean score of 2 indicates a person that on average responds ‘Agree.’

### Risk factor analysis

**Fig. 1:**
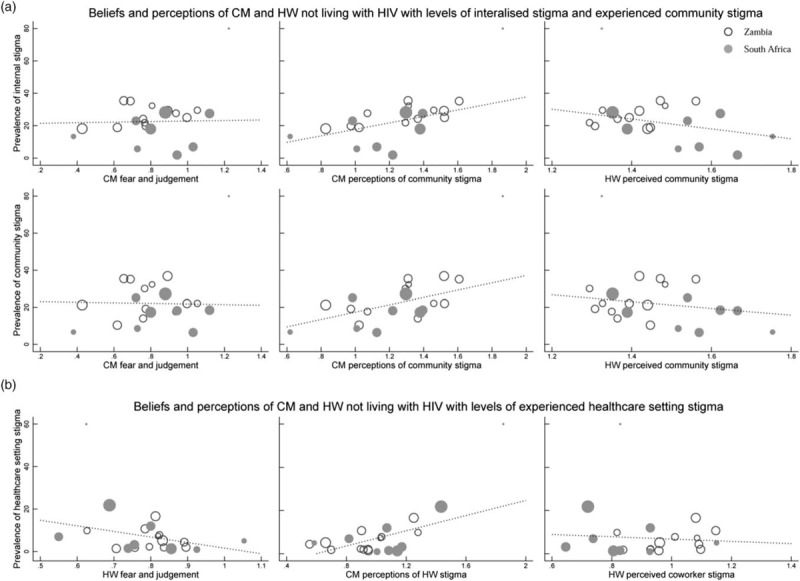
Cluster-level scatter plots showing the association between the beliefs and perceptions of community members and health workers not living with HIV and levels of (a) internalized stigma and experienced community stigma, and (b) experienced healthcare setting stigma reported by people living with HIV in 21 communities in South Africa and Zambia.

Internalized stigma was not significantly associated with sociodemographic or behavioural characteristics, except that it was reported less often by those who had been diagnosed for longer (aOR 0.75, 95% CI 0.59–0.96 and aOR 0.73, 95% CI 0.56–0.96, comparing 1–5 years and more than 5 years since diagnosis with 0–12 months, respectively). There was some evidence of more internalized stigma reported by those of higher wealth (*P* = 0.065). Internalized stigma was more commonly reported by those reporting stigma experienced in both community and healthcare settings (aOR 4.32, 95% CI 3.47–5.37 and aOR 4.37, 95% CI 2.71–7.06, respectively; Table 4). Internalized stigma was not significantly associated with living in a community with a higher score for HIV fear and judgement held by CM (adjusted odds ratio for a unit increase in the score, aOR_score_ 1.11, 95% CI 0.36–3.44). However, internalized stigma was significantly associated with the average level of perceived stigma reported by CM (aOR_score_ 3.36, 95% CI 1.86–6.10). There was little evidence of an association between internalized stigma and the HWs’ perceptions of the level of stigma in the community (aOR_score_ 0.16, 95% CI 0.01–2.34; Table 5). These findings were mirrored in the cluster-level scatter plots (Fig. 1).

**Table 4:** The association between sociodemographic and behavioural characteristics and three types of stigma among 3859 people living with HIV from 21 study communities in Zambia and South Africa.

		Any internalized stigma			Stigma experienced in the community			Stigma experiences in a healthcare setting		
Variable	Categories	*n*/*N* (%)	aOR (95% CI)^a^	*P*_w_^b^	*n*/*N* (%)	aOR (95% CI)^a^	*P*_w_^b^	*n*/*N* (%)	aOR (95% CI)^a^	*P*_w_^b^
Sex	Male	108/456 (23.7%)	1.00	0.136	92/456 (20.2%)	1.00	0.016	23/456 (5.0%)	1.00	0.019
	Female	760/3403 (22.3%)	0.90 (0.78–1.03)		761/3403 (22.4%)	1.22 (1.04–1.43)		257/3403 (7.6%)	1.64 (1.08–2.48)	
Age	<24	111/423 (26.2%)	1.00	0.154	74/423 (17.5%)	1.00	0.011	17/423 (4.0%)	1.00	0.086
	25–34	402/1777 (22.6%)	0.82 (0.64–1.05)		370/1777 (20.8%)	1.25 (0.93–1.68)		130/1777 (7.3%)	1.91 (0.96–3.82)	
	35–44	355/1659 (21.4%)	0.75 (0.56–1.01)		409/1659 (24.7%)	1.58 (1.15–2.17)		133/1659 (8.0%)	2.19 (1.07–4.48)	
Education	Did not complete secondary	334/1344 (24.9%)	1.00	0.277	326/1344 (24.3%)	1.00	0.063	78/1344 (5.8%)	1.00	0.352
	Completed secondary	500/2360 (21.2%)	0.81 (0.62–1.05)		483/2360 (20.5%)	0.82 (0.62–1.07)		191/2360 (8.1%)	1.45 (0.87–2.42)	
	Further	34/155 (21.9%)	0.84 (0.59–1.18)		44/155 (28.4%)	1.28 (0.91–1.80)		11/155 (7.1%)	1.32 (0.69–2.53)	
Marital status	Not married	456/2005 (22.7%)	1.00	0.881	483/2005 (24.1%)	1.00	0.009	160/2005 (8.0%)	1.00	0.349
	Married	412/1854 (22.2%)	0.98 (0.80–1.21)		370/1854 (20.0%)	0.77 (0.63–0.94)		120/1854 (6.5%)	0.77 (0.45–1.32)	
Ever started ART	No	68/340 (20.0%)	1.00	0.631	70/340 (20.6%)	1.00	0.416	27/340 (7.9%)	1.00	0.792
	Yes	590/2690 (21.9%)	1.14 (0.67–1.94)		645/2690 (24.0%)	1.19 (0.78–1.81)		197/2690 (7.3%)	0.89 (0.39–2.05)	
	Do not know	210/829 (25.3%)	–		138/829 (16.6%)			56/829 (6.8%)	–	
Disclosed to (ever disclosed HIV status)	No	72/300 (24.0%)	1.00	0.578	38/300 (12.7%)	1.00	<0.001	16/300 (5.3%)	1.00	0.379
	Yes	796/3559 (22.4%)	0.93 (0.71–1.21)		815/3559 (22.9%)	1.99 (1.51–2.63)		264/3559 (7.4%)	1.37 (0.68–2.74)	
How long has it been since your first positive HIV test?	0–11 months	212/750 (28.3%)	1.00	0.043	115/750 (15.3%)	1.00	<0.001	36/750 (4.8%)	1.00	0.097
	1–5 years	351/1543 (22.7%)	0.75 (0.59–0.96)		342/1543 (22.2%)	1.51 (1.20–1.89)		106/1543 (6.9%)	1.36 (0.85–2.18)	
	More than 5 years	167/754 (22.1%)	0.73 (0.56–0.96)		215/754 (28.5%)	2.04 (1.59–2.63)		60/754 (8.0%)	1.53 (1.02–2.30)	
	Skipped/missing	138/812 (17.0%)	–		181/812 (22.3%)			78/812 (9.6%)	–	
How old were you the first time you had sex	11–15	157/632 (24.8%)	1.00	0.527	158/632 (25.0%)	1.00	0.361	45/632 (7.1%)	1.00	0.477
	16–18	421/1854 (22.7%)	0.91 (0.69–1.20)		414/1854 (22.3%)	0.83 (0.63–1.09)		139/1854 (7.5%)	1.00 (0.50–1.98)	
	19–24	213/964 (22.1%)	0.88 (0.63–1.24)		215/964 (22.3%)	0.81 (0.62–1.05)		81/964 (8.4%)	1.10 (0.57–2.13)	
	25+	8/50 (16.0%)	0.59 (0.30–1.19)		13/50 (26.0%)	0.99 (0.48–2.03)		1/50 (2.0%)	0.25 (0.03–2.42)	
	Skipped/missing	69/359 (19.2%)	–		53/359 (14.8%)			14/359 (3.9%)	–	
How many sexual partners have you had in your lifetime?	1	120/639 (18.8%)	1.00	0.107	108/639 (16.9%)	1.00	0.002	39/639 (6.1%)	1.00	0.001
	2–5	520/2156 (24.1%)	1.38 (0.90–2.13)		518/2156 (24.0%)	1.55 (0.94–2.55)		168/2156 (7.8%)	1.29 (0.57–2.91)	
	6–10	88/410 (21.5%)	1.17 (0.68–2.02)		107/410 (26.1%)	1.76 (0.98–3.18)		32/410 (7.8%)	1.37 (0.40–4.72)	
	11–15	12/52 (23.1%)	1.27 (0.69–2.37)		17/52 (32.7%)	2.46 (1.14–5.28)		7/52 (13.5%)	2.64 (0.86–8.15)	
	16–20	7/28 (25.0%)	1.43 (0.54–3.75)		11/28 (39.3%)	3.24 (1.40–7.53)		5/28 (17.9%)	3.71 (0.61–22.63)	
	More than 20	16/40 (40.0%)	2.73 (1.19–6.26)		17/40 (42.5%)	3.91 (1.80–8.49)		4/40 (10.0%)	2.24 (0.91–5.49)	
	Skipped/missing	105/534 (19.7%)	–		75/534 (14.0%)			25/534 (4.7%)	–	
The last time you had sex, did you use a condom	No	250/1001 (25.0%)	1.00	0.323	236/1001 (23.6%)	1.00	0.187	66/1001 (6.6%)	1.00	0.604
	Yes	413/1857 (22.2%)	0.86 (0.65–1.15)		394/1857 (21.2%)	0.87 (0.71–1.07)		137/1857 (7.4%)	1.11 (0.74–1.66)	
	Skipped/missing	205/1001 (20.5%)	–		223/1001 (22.3%)			77/1001 (7.7%)	–	
Wealth tertile	Lowest	318/1547 (20.6%)	1.00	0.065	307/1547 (19.8%)	1.00	0.197	88/1547 (5.7%)	1.00	0.003
	Middle	387/1583 (24.4%)	1.26 (1.03–1.55)		364/1583 (23.0%)	1.19 (0.94–1.51)		116/1583 (7.3%)	1.28 (0.99–1.66)	
	Highest	163/729 (22.4%)	1.12 (0.84–1.51)		182/729 (25.0%)	1.32 (0.96–1.80)		76/729 (10.4%)	1.90 (1.30–2.79)	
Stigma experienced in the community	No	486/3006 (16.2%)	1.00	<0.001	–	–		–	–	
	Yes	382/853 (44.8%)	4.32 (3.47–5.37)		–	–		–	–	
Stigma experienced in a healthcare setting	No	723/3579 (20.2%)	1.00	<0.001	616/3579 (17.2%)	1.00	<0.001	–	–	
	Yes	145/280 (51.8%)	4.37 (2.71–7.06)		237/280 (84.6%)	26.28 (13.22–52.26)		–	–	

aOR, adjusted odds ratio; CI, confidence interval; *n*, number of individuals experiencing the three types of stigma within groups; *N*, total number of individuals within groups; *P*_w_, *P* value of the Wald test.

^a^The aOR for sex is adjusted for age group; the aOR for age group is adjusted for sex; the aOR for all other predictor variables are adjusted for sex and age group.

^b^A *P* value of less than 0.05 indicates that the predictor creates a statistically significant improvement in the fit of the model.

**Table 5:** Association between attitudes and perceptions of stigma held by community members and health workers and levels of internalized and experienced stigma reported by people living with HIV adjusted for age, sex and clustering (*n* = 3859).

	Any internalized stigma	Any experienced stigma	Stigma experience in a healthcare setting
Cluster-level exposure variables	aOR_score_ (95% CI) for unit increase in score	aOR_score_ (95% CI) for unit increase in score	aOR_score_ (95% CI) for unit increase in score
Average level of HIV fear and judgement reported by CM	1.11 (0.36–3.44)	0.89 (0.31–2.58)	–
Average level of perceived HIV stigma in the community reported by CM	3.36 (1.86–6.10)	3.27 (1.31–8.19)	–
Average level of perceived HIV stigma in healthcare settings reported by CM	–	–	14.93 (3.95–56.43)
Average level of HIV fear and judgement reported by HW	–	–	0.02 (0.01–1.60)
Average level of perceived HIV stigma in the community reported by HW	0.16 (0.01–2.34)	0.34 (0.07–1.71)	–
Average level of perceived co-worker stigmatizing behaviour reported by HW	–	–	0.49 (0.01–32.0)

aOR_score_, adjusted odds ratio for age, sex and clustering within communities; CI, confidence interval; CM, community members not living with HIV; HW, health workers not living with HIV.

Each circle represents one cluster. Size of the circles are proportional to the number of PLHIV respondents in each cluster. Dotted lines reflect linear regression slopes from cluster-level analyses of the associations and weighted by the size of the PLHIV community in each cluster. CM fear and judgement, Average level of HIV fear and judgement reported by community members; CM perceptions of community stigma, average level of perceived stigma in the community reported by community members; CM perceptions of HW stigma, average level of perceived stigma in healthcare settings reported by community members; CM, community member; HW fear and judgement, average level of fear and judgement reported by health workers; HW perceived community stigma, average level of perceived stigma in the community reported by health workers; HW perceived co-worker stigma, average level of perceived co-workers stigmatizing behaviour reported by health workers; HW, health worker; PlHIV, people living with HIV.

Stigma experienced in the community was more frequently reported by women than men (aOR 1.22, 95% CI 1.04–1.43), older individuals (aOR 1.58, 95% CI 1.15–2.17 comparing 35–44 with ≤24-year olds) and those who were currently unmarried (aOR 0.77, 95% CI 0.63–0.94 comparing married with unmarried). This form of stigma was more commonly reported by those who had disclosed their HIV status (aOR 1.99, 95% CI 1.51–2.63), and had been diagnosed longer ago (aOR 2.04, 95% CI 1.59–2.63 comparing 5+ years with 0–12 months since diagnosis), as well as those reporting more lifetime sexual partners (e.g. aOR 3.91, 95% CI 1.80–8.49 comparing >20 partners with one partner in lifetime). Stigma was more commonly experienced in the community among individuals who had also experienced stigma in a healthcare setting (aOR 26.28, 95% CI 13.22–52.26; Table 4). The proportion of PLHIV experiencing stigma in the community was not associated with living in a community with a higher score for fear and judgement in CMs’ attitudes (aOR_score_ 0.89, 95% CI 0.31–2.58), but was associated with the level of perceived stigma in the community reported by CM (aOR_score_ 3.27, 95% CI 1.31–8.19). There was little evidence of an association between community-experienced stigma and HW's perceptions of stigma in communities (aOR_score_ 0.34, 95% CI 0.07–1.71) (Table 5).

Stigma experienced in a healthcare setting was more commonly reported by women than men (aOR 1.64, 95% CI 1.08–2.48), and among those reporting more lifetime sexual partners (e.g. aOR 2.24, 95% CI 0.91–5.49 comparing >20 partners with those with one partner; Table 4). Odds ratios for the associations between community level characteristics and stigma experienced in a health setting had wide confidence intervals (Table 5). Despite this, there was evidence of an association such that CMs’ perceptions that stigma was present in healthcare settings was associated with PLHIV reports of this (aOR_score_ 14.93, 95% CI 3.95–56.43).

## Discussion

In this large study in 21 urban communities across two countries, 35.5% of PLHIV reported some type of stigma. Most PLHIV participants were women, reflecting both higher response rates and a higher prevalence of HIV among women. Individuals reporting one form of stigma were more likely to report the other types. Experienced stigma in the community and internalized stigma were more common in Zambian communities, whereas experienced stigma in healthcare settings was more common in South African communities. There were few individual predictors of internalized stigma, but experienced stigma was associated with sociodemographic and behavioural characteristics. At cluster level, community members’ (but not HW) perceptions of stigma varied substantially across communities and were associated with PLHIV experiences. However, surprisingly, CMs’ reported attitudes of fear and judgement toward PLHIV were not associated with PLHIV's reported experiences of stigma.

We have undertaken the largest ever study on experiences of stigma from a random sample of PLHIV, adopting best-practice measures of core manifestations of HIV stigma [7,12,15]. Although some PLHIV did not participate in the study or did not disclose their status, response rates were high. We have brought data from PLHIV together with independently collected data on the beliefs and perceptions of HIV stigma held by community members and HW. These fears, judgements and perceptions are thought to act as drivers of stigma in communities [19]. This ‘parallel’ approach to data collection has been discussed in the literature [11,15,16] but not operationalized. Aside from the strengths of our work, there are also limitations. Stigma is a sensitive subject and may have been under reported. Social desirability bias might have affected the validity of responses to beliefs and perception questions [20,21]. As the communities involved in the study were purposively selected it is unclear how generalizable our findings are to other settings in sub-Saharan Africa. Wide confidence intervals for some associations reflect few events for some outcomes, limited intercluster variation for some exposure variables and the small number of clusters [22]. Finally, results for risk factors with missing data should be interpreted with caution noting that PLHIV with missing data were less likely to report HIV stigma.

Reported experience of stigma among PLHIV in our study was lower than in studies employing the PLHIV Stigma index in South Africa [23] and Zambia [24]. In 2009, 51.8% of PLHIV reported having experienced verbal abuse because of their HIV status in Zambia compared to 8.3% in our study [24]. In another study, 16.1% of PLHIV reported physical abuse because of their HIV status compared to 4.6% in our population [19]. Previous studies were conducted on smaller, convenience or snowball samples [25]. Individuals recruited this way may not be representative of all PLHIV [26]. Participants may be more likely to discuss stigma in the PLHIV Stigma Index studies as these are partly used to encourage reflection on life experiences living with HIV [19,23,24]. Our findings are consistent with the hypothesis that some forms of stigma may be decreasing over time and as ART access expands [27]. Stigma manifestations may also be shifting with more nuanced forms of stigma replacing overt acts of stigma and discrimination [28].

Some findings were as hypothesized. Experienced stigma was more common among those reporting more risk behaviour. Those who had been diagnosed for longer and who had disclosed to others reported more experienced stigma, perhaps reflecting their greater visibility [29,30]. They also reported less internalized stigma, perhaps reflecting having had a longer period to ‘accept’ their status [31–33]. Other findings were unexpected. Although community members’ perceptions of levels of stigma were correlated with the reported experiences of PLHIV, neither their beliefs, nor the beliefs or perceptions of HW were. This may reflect misreporting of either stigma experiences, or of beliefs, because of social desirability bias [20,21]. However, our study used electronic data collection devices and sought to encourage honest reporting. Stigma reported by PLHIV might also have occurred outside the study communities or healthcare settings from which belief data were collected. Nevertheless, our results suggest caution in situations wherever reported fears and judgements are interpreted as a proxy for the experiences of PLHIV. It also underscores the role of internalized stigma in contributing to stigma experiences.

Stigma remains an important phenomenon in these study communities. Our results will inform ongoing work addressing the core hypotheses for our nested study: that the HPTN 071 (PopART) intervention may reduce levels of stigma in study communities, that stigma may undermine the effectiveness of efforts to scale up testing and treatment, or that the forms of HIV stigma may change over the period of the trial [15].

## Acknowledgements

Funding: HPTN 071 (PopART) is sponsored by the National Institute of Allergy and Infectious Diseases (NIAID) under Cooperative Agreements UM1-AI068619, UM1-AI068617 and UM1-AI068613, with funding from the US President's Emergency Plan for AIDS Relief (PEPFAR). Additional funding is provided by the International Initiative for Impact Evaluation (3ie) with support from the Bill & Melinda Gates Foundation, as well as by NIAID, the National Institute on Drug Abuse (NIDA) and the National Institute of Mental Health (NIMH), all part of NIH. The content is solely the responsibility of the authors and does not necessarily represent the official views of the NIAID, NIMH, NIDA, PEPFAR, 3ie or the Bill & Melinda Gates Foundation.

J.R.H, A.S. and S.K. are members of the STRIVE consortium, which produces research on the structural drivers of HIV, including stigma. The STRIVE consortium is funded by UKaid from the Department for International Development (http://strive.lshtm.ac.uk/). However, the views expressed do not necessarily reflect the department's official policies.

Author contributions: J.R.H. conceived the analysis and led the writing of the article. S.K. undertook the analysis and assisted with the delivery of the study in the field. H.M. and P.L. contributed to the analysis. K.S. undertook literature review. H.M., N.M. and T.M. led delivery of the study in the field. A.Sc. and R.V. oversaw data management and quality assurance. D.D., H.A. and R.J. designed and led the cluster-randomized trial and population cohort study within which the study is nested. E.P.-M. oversaw the laboratory testing. G.H. and V.B. were responsible for the in-country management including data collection and with A.St. designed the questions on stigma included in this analysis and are co-investigators on the study protocol. All authors contributed to the writing of the article and have agreed the final draft for submission.

We thank Triantafyllos Pliakas for his help on the statistical analysis and Ranjeeta Thomas who undertook the asset index analysis as an index of wealth.

### Conflicts of interest

There are no conflicts of interest.

## Supplementary Material

Supplemental Digital Content
