# Supplementary material for: Individual and community-level risk factors for HIV stigma in 21 Zambian and South African communities: analysis of data from the HPTN071 (PopART) study
Source: AIDS. 2018 Mar 21;32(6):783–93. doi: 10.1097/QAD.0000000000001757 (PMC5854764; doi:10.1097/QAD.0000000000001757)
Supplement: Supplemental Digital Content [file aids-32-783-s001.docx]

**Appendix S.1: Sources and types of outcome and exposure data used in the analysis**

| **Use of data in the analysis** | **Source of data** | **Sub-population** | **Specific Variables** | **Form of data use in analysis** | **N** |
| --- | --- | --- | --- | --- | --- |
| Outcomes | Population Cohort | PLHIV  People living with HIV (self-report and laboratory confirmed) | Internalised stigma^1^ | Binary outcomes | 3859 |
|  |  |  | Stigma experienced in the community^2^ |  |  |
|  |  |  | Stigma experienced in a healthcare setting^3^ |  |  |
| Individual-level exposures | Population Cohort | PLHIV  People living with HIV (self-report and laboratory confirmed) | Sex, Age, Education, Marital Status, Ever started ART, Disclosure, Time since diagnosis, Sexual Behaviour Characteristics, Household Wealth* | Categorical exposures | 3859 |
| Cluster-level exposures | Population Cohort | CM  Community members not living with laboratory confirmed HIV | Average level of HIV fear and judgement reported by community members^4^ | Continuous exposures, with possible variation from 0-3 | 5088 |
|  |  |  | Average level of perceived stigma in the community  reported by community members^5^ |  |  |
|  |  |  | Average level of perceived stigma in healthcare settings reported by community members^6^ |  |  |
|  | Health worker Cohort | HW  Health Facility Staff and community health workers self-reporting not to be living with HIV | Average level of HIV fear and judgement reported by health workers^7^ | Continuous exposures, with possible variation from 0-3 | 851 |
|  |  |  | Average level of perceived stigma in the community reported by health workers ^8^ |  |  |
|  |  |  | Average level of perceived co-workers stigmatising behaviour reported by health workers ^9^ |  |  |

nLWH: not living with HIV.

Questions asked in the creation of each score are as follows. All the questions below were asked on a four point Likert scale (Strongly Disagree, Disagree, Agree, Strongly Agree) except 2&3 which were asked on a frequency Likert (Never, Once, A few Times, Often)

^1^Three items: I think less of myself because of my HIV status; I have felt ashamed because of my HIV status; I have lost respect or standing in the community because of my HIV status

^2^Five items People have talked badly about me because of my HIV status; I have been verbally insulted, harassed and/or threatened because of my HIV status; I have been physically assaulted because of my HIV status; Someone else disclosed my HIV status without my permission; I have felt that people have not wanted to sit next to me, for example on public transport, at church or in the waiting room because of my HIV

^3^ Three items; I have been denied health services because of my HIV status; Healthcare workers talked badly about me because of my HIV status; A health worker disclosed my HIV status without my permission

^4^Three items: I fear that I could contract HIV if I come into contact with the saliva of a person living with HIV; I would not like to sit close to someone living with HIV, for example on public transport, at church or in a waiting room; I would be ashamed if someone in my family had HIV

^5^Five items: People thought to be living with HIV are sometimes physically assaulted; People sometimes talk badly about PLHIV to others; People thought to be living with HIV lose respect or standing; People thought to be living with HIV are verbally insulted, harassed, or threatened; People sometimes disclose that other people are HIV positive without their permission

^6^Two items: Health workers sometimes talk badly about people living with or thought to be living with HIV to others; Health workers sometimes disclose that other people are HIV positive without their permission

^7^Five items: I fear that I could contract HIV if I come into contact with the saliva of a person living with HIV; I avoid physical contact with clients living with HIV; HIV is punishment from God; Other people deserve access to health services more than PLHIV; I would be ashamed if someone in my family had HIV

^8^Five items: People thought to be living with HIV are sometimes physically assaulted; People sometimes talk badly about PLHIV to others; People thought to be living with HIV lose respect or standing; People thought to be living with HIV are verbally insulted, harassed, or threatened; People hesitate to start ARV drugs because they are afraid others will learn they are living with HIV

^9^Four items: My co-workers sometimes talk badly about people thought to be living with HIV; My co-workers sometimes gossip about clients' HIV test results; My co-workers sometimes treat people living with HIV poorly when providing them with health services; My co-workers sometimes verbally insult clients living with HIV

* Asset index generated from principal components analysis of data on the following assets: materials used to construct house walls, type of floor, water source, energy source for home, sanitation facilities, and ownership of: cell phone, bicycle, motorcycle/scooter, car, household electricity, television, fridge/freezer, radio, computer, and/or music playing device. Principal components analysis to derive weights was run separately for each country, then tertiles created, before combining the data across countries.


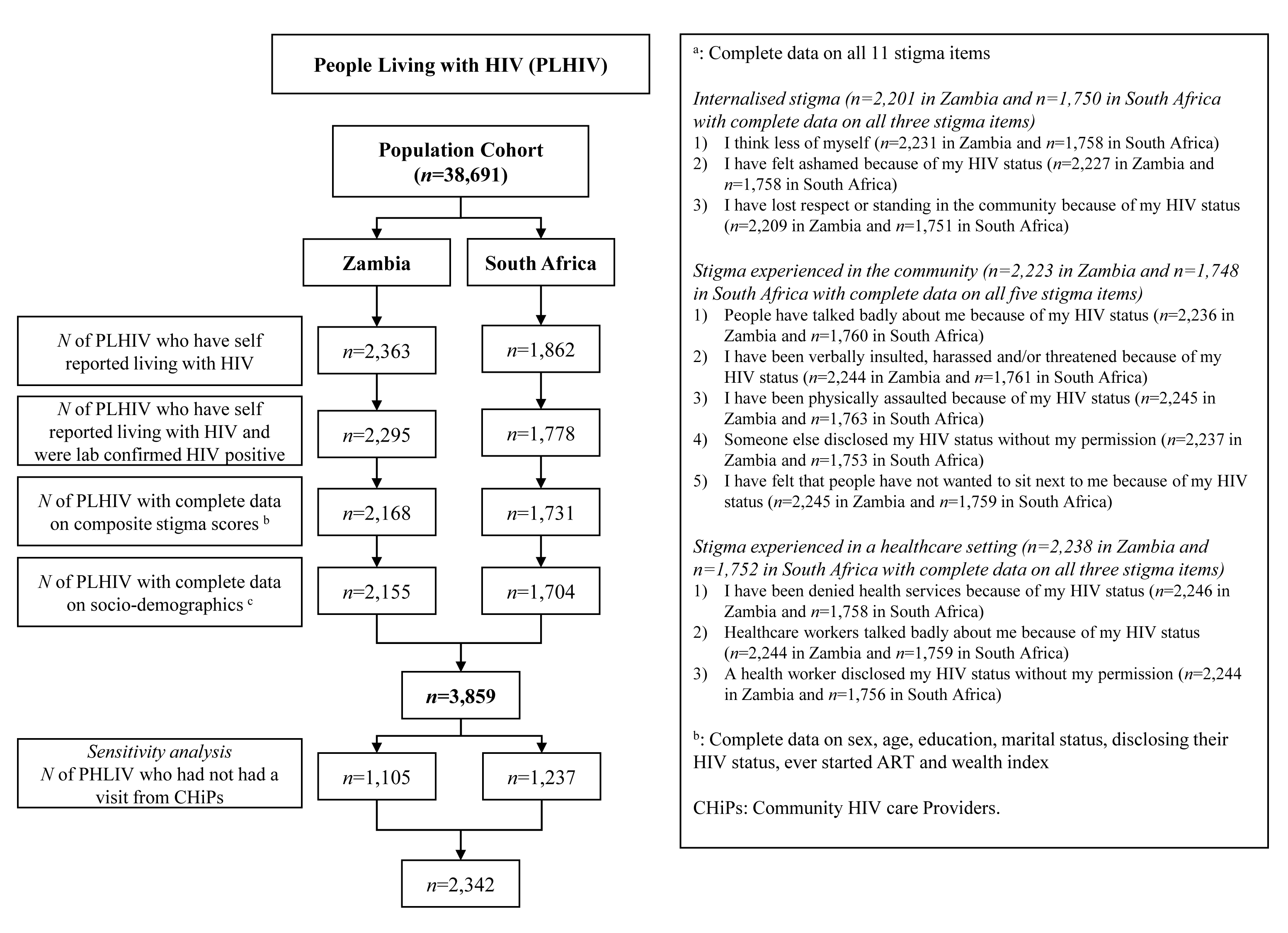


**Figure S.1a.** Flowchart for people living with HIV (self-report and laboratory confirmed) (**PLHIV**).

**
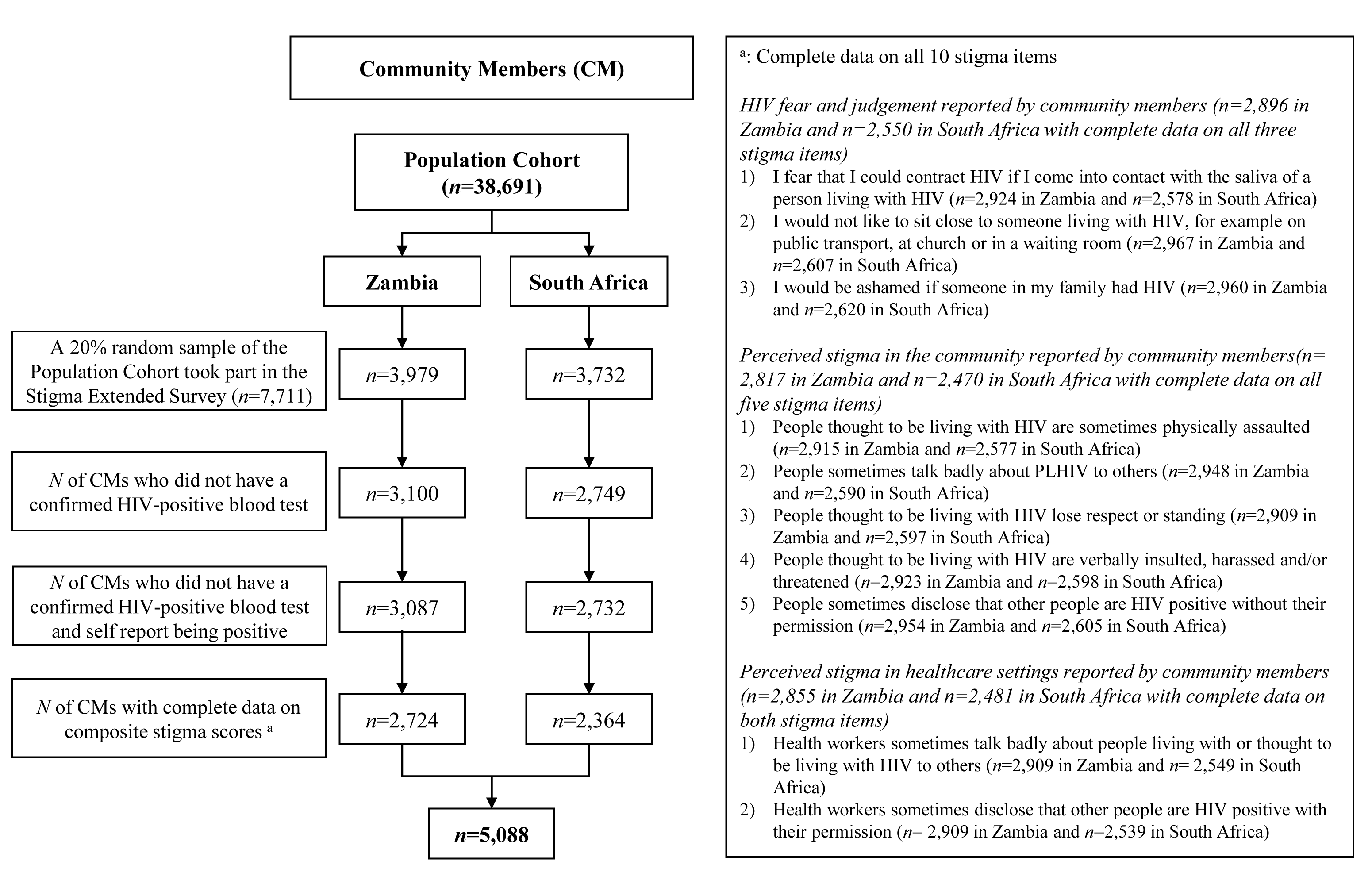
**

**Figure S.1b.** Flowchart for community members not living with laboratory confirmed HIV (**CM**).


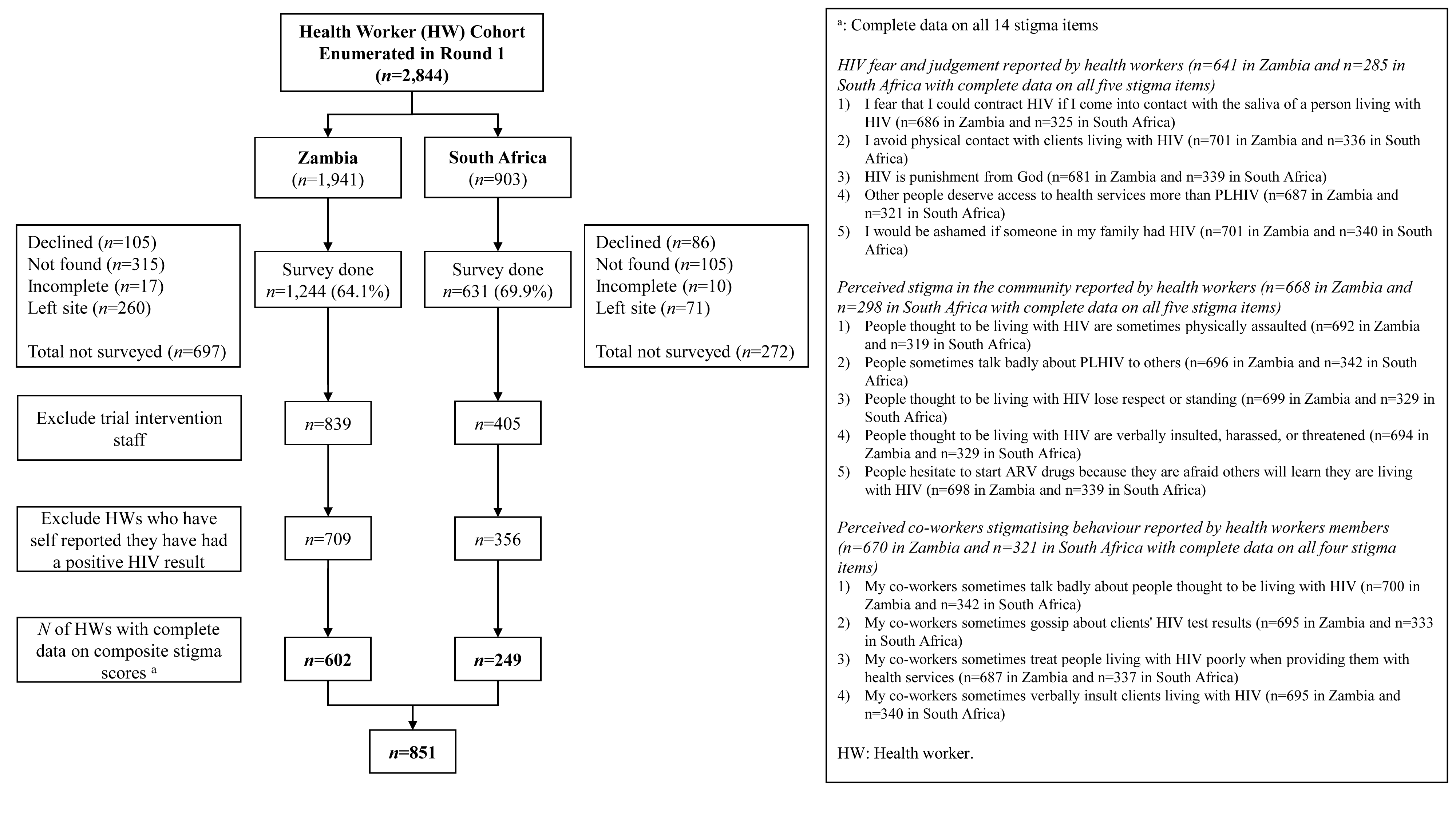


**Figure S.1c.** Flowchart for the dataset derived from the health worker cohort; Health Facility Staff and community health workers self-reporting not to be living with HIV (**HW**).

**Appendix S2.** Sensitivity analysis of 2,342 PLHIV who had not yet had a visit from community HIV care providers.

The table below reflects the same analysis as shown in Table 3 of the main paper. However, this analysis is restricted to PLHIV who, at the time of interview, had not yet been visited by a PopART intervention community health worker. Our sensitivity analysis that the prevalence of stigma was largely unchanged and while there were changes in point estimates and significance values for individual variables there were no systematic differences of interpretation.

Internalised stigma was reported less often by older individuals (aOR 0.70 95% CI 0.53-0.93 comparing 25-34 with <=24 year olds), those who were more educated (aOR 0.74 95% CI 0.56-0.98 and aOR 0.65 95% CI 0.47-0.91 comparing those who completed secondary and further education, respectively, with those who did not complete secondary education) and those who used a condom the last time they had sex (aOR 0.70 95% CI 0.54-0.90). We found no difference in terms of how long individuals have been diagnosed with HIV. Similarly to the results of the main analysis, internalised stigma was more commonly reported by those reporting stigma experienced in both community and healthcare settings.

Stigma experienced in the community was less often reported by those who used a condom the last time they had sex (aOR 0.79 95% CI 0.64-0.97) and there was some evidence of more stigma reported by those with a higher number of sexual patterns (for example, aOR 3.01 95% CI 1.21-7.51 comparing >20 partners with 1 partner in lifetime). We found no difference in terms of marital status. Similarly to the results of the main analysis, stigma experienced in the community was more frequently reported by women than men, older individuals, those who had disclosed their HIV status and those who had been diagnosed longer ago and among individuals who had also experienced stigma in a healthcare setting.

We found no difference between men and women in reporting stigma experienced in a healthcare setting. Similarly to the results of the main analysis, stigma experienced in a healthcare setting was more common among those reporting more lifetime sexual partners (for example, aOR 2.48 95% CI 0.78-7.92 comparing >20 partners with 1 partner in lifetime) and more wealth (aOR 1.31 95% CI 0.96-1.77 and aOR 1.80 95% CI 1.23-2.64 comparing those from the middle and highest tertile, respectively, with those from the lowest tertile).

Appendix S2. The association between sociodemographic and behavioural characteristics and three types of stigma among 2342 PLHIV from 21 study communities who didn’t have a visit from a community HIV care provider (CHiP) in Zambia and South Africa.

|  |  | **Any internalised stigma** | | | **Stigma experienced in the community** | | | **Stigma experienced in a healthcare setting** | | |
| --- | --- | --- | --- | --- | --- | --- | --- | --- | --- | --- |
| Variable | Categories | **n/N (%)** | **aOR (95% CI)^a^** | **P_w_^b^** | **n/N (%)** | **aOR (95% CI)^a^** | **P_w_^b^** | **n/N (%)** | **aOR (95% CI)^a^** | **P_w_^b^** |
| Sex | Male | 73/289 (25.3%) | 1.00 | 0.402 | 54/289 (18.7%) | 1.00 | 0.001 | 17/289 (5.9%) | 1.00 | 0.128 |
|  | Female | 489/2053 (23.8%) | 0.89 (0.68-1.17) |  | 487/2053 (23.7%) | 1.44 (1.15-1.81) |  | 167/2053 (8.1%) | 1.51 (0.89-2.56) |  |
| Age | <24 | 77/254 (30.3%) | 1.00 | 0.045 | 48/254 (18.9%) | 1.00 | 0.014 | 11/254 (4.3%) | 1.00 | 0.089 |
|  | 25-34 | 252/1073 (23.5%) | 0.70 (0.53-0.93) |  | 230/1073 (21.4%) | 1.19 (0.81-1.76) |  | 85/1073 (7.9%) | 1.93 (0.82-4.57) |  |
|  | 35-44 | 233/1015 (23.0%) | 0.68 (0.45-1.01) |  | 263/1015 (25.9%) | 1.57 (1.08-2.28) |  | 88/1015 (8.7%) | 2.19 (1.00-4.83) |  |
| Education | Did not complete secondary | 203/731 (27.8%) | 1.00 | 0.019 | 190/731 (26.0%) | 1.00 | 0.109 | 55/731 (7.5%) | 1.00 | 0.512 |
|  | Completed secondary | 338/1508 (22.4%) | 0.74 (0.56-0.98) |  | 323/1508 (21.4%) | 0.79 (0.63-0.98) |  | 124/1508 (8.2%) | 1.12 (0.72-1.73) |  |
|  | Further | 21/103 (20.4%) | 0.65 (0.47-0.91) |  | 28/103 (27.2%) | 1.13 (0.76-1.68) |  | 5/103 (4.9%) | 0.66 (0.31-1.40) |  |
| Marital status | No | 312/1272 (24.5%) | 1.00 | 0.723 | 311/1272 (24.4%) | 1.00 | 0.101 | 102/1272 (8.0%) | 1.00 | 0.743 |
|  | Yes | 250/1070 (23.4%) | 0.96 (0.78-1.18) |  | 230/1070 (21.5%) | 0.82 (0.65-1.04) |  | 82/1070 (7.7%) | 0.91 (0.54-1.56) |  |
| Ever started ART | No | 51/218 (23.4%) | 1.00 | 0.988 | 51/218 (23.4%) | 1.00 | 0.806 | 21/218 (9.6%) | 1.00 | 0.514 |
|  | Yes | 365/1598 (22.8%) | 1.00 (0.60-1.67) |  | 397/1598 (24.8%) | 1.06 (0.67-1.66) |  | 123/1598 (7.7%) | 0.75 (0.31-1.79) |  |
|  | Don't know | 146/526 (27.8%) |  |  | 93/526 (17.7%) |  |  | 40/526 (7.6%) |  |  |
| Disclosed to (ever disclosed HIV status) | No | 44/183 (24.0%) | 1.00 | 0.854 | 25/183 (13.7%) | 1.00 | <0.001 | 12/183 (6.6%) | 1.00 | 0.739 |
|  | Yes | 518/2159 (24.0%) | 1.03 (0.76-1.38) |  | 516/2159 (23.9%) | 1.91 (1.43-2.54) |  | 172/2159 (8.0%) | 1.17 (0.47-2.92) |  |
| How long has it been since your first positive HIV test? | 0-11 months | 126/424 (29.7%) | 1.00 | 0.248 | 73/424 (17.2%) | 1.00 | <0.001 | 26/424 (6.1%) | 1.00 | 0.344 |
|  | 1-5 years | 242/959 (25.2%) | 0.82 (0.60-1.10) |  | 220/959 (22.9%) | 1.36 (1.02-1.81) |  | 73/959 (7.6%) | 1.17 (0.70-1.97) |  |
|  | More than 5 years | 111/471 (23.6%) | 0.74 (0.53-1.05) |  | 140/471 (29.7%) | 1.85 (1.43-2.38) |  | 42/471 (8.9%) | 1.35 (0.83-2.18) |  |
|  | Skipped/missing | 83/488 (17.0%) |  |  | 108/488 (22.1%) |  |  | 43/488 (8.8%) |  |  |
| How old were you the first time you had sex | 11-15 | 101/399 (25.3%) | 1.00 | 0.957 | 98/399 (24.6%) | 1.00 | 0.340 | 28/399 (7.0%) | 1.00 | 0.543 |
|  | 16-18 | 273/1152 (23.7%) | 0.96 (0.70-1.31) |  | 266/1152 (23.1%) | 0.87 (0.61-1.23) |  | 93/1152 (8.1%) | 1.08 (0.48-2.41) |  |
|  | 18-24 | 140/569 (24.6%) | 1.02 (0.69-1.51) |  | 132/569 (23.2%) | 0.85 (0.61-1.20) |  | 53/569 (9.3%) | 1.23 (0.61-2.48) |  |
|  | 25+ | 6/28 (21.4%) | 0.87 (0.35-2.17) |  | 9/28 (32.1%) | 1.30 (0.52-3.25) |  | 1/28 (3.6%) | 0.43 (0.04-4.74) |  |
|  | Skipped/missing | 42/194 (21.6%) |  |  | 36/194 (18.6%) |  |  | 9/194 (4.6%) |  |  |
| How many sexual partners have you had in your lifetime? | 1 | 84/384 (21.9%) | 1.00 | 0.426 | 71/384 (18.5%) | 1.00 | 0.054 | 31/384 (8.1%) | 1.00 | 0.001 |
|  | 2-5 | 327/1298 (25.2%) | 1.21 (0.73-2.00) |  | 320/1298 (24.7%) | 1.45 (0.82-2.55) |  | 113/1298 (8.7%) | 1.08 (0.48-2.42) |  |
|  | 6-10 | 64/284 (22.5%) | 1.02 (0.55-1.92) |  | 77/284 (27.1%) | 1.71 (0.86-3.39) |  | 19/284 (6.7%) | 0.85 (0.25-2.86) |  |
|  | 11-15 | 5/26 (19.2%) | 0.83 (0.24-2.89) |  | 9/26 (34.6%) | 2.43 (1.08-5.48) |  | 3/26 (11.5%) | 1.58 (0.60-4.16) |  |
|  | 16-20 | 3/17 (17.6%) | 0.75 (0.23-2.40) |  | 6/17 (35.3%) | 2.75 (0.98-7.68) |  | 3/17 (17.6%) | 2.74 (0.32-23.63) |  |
|  | More than 20 | 11/27 (40.7%) | 2.25 (0.88-5.80) |  | 10/27 (37.0%) | 3.01 (1.21-7.51) |  | 4/27 (14.8%) | 2.48 (0.78-7.92) |  |
|  | Skipped/missing | 68/306 (22.2%) |  |  | 48/306 (15.7%) |  |  | 11/306 (3.6%) |  |  |
| The last time you had sex, did you use a condom | No | 165/559 (29.5%) | 1.00 | 0.006 | 144/559 (25.8%) | 1.00 | 0.025 | 47/559 (8.4%) | 1.00 | 0.563 |
|  | Yes | 268/1201 (22.3%) | 0.70 (0.54-0.90) |  | 260/1201 (21.6%) | 0.79 (0.64-0.97) |  | 91/1201 (7.6%) | 0.87 (0.55-1.39) |  |
|  | Skipped/missing | 129/582 (22.2%) |  |  | 137/582 (23.5%) |  |  | 46/582 (7.9%) |  |  |
| Wealth Tertile | Lowest | 195/889 (21.9%) | 1.00 | 0.360 | 184/889 (20.7%) | 1.00 | 0.186 | 55/889 (6.2%) | 1.00 | 0.006 |
|  | Middle | 252/984 (25.6%) | 1.23 (0.93-1.64) |  | 232/984 (23.6%) | 1.16 (0.82-1.64) |  | 79/984 (8.0%) | 1.31 (0.96-1.77) |  |
|  | Highest | 115/469 (24.5%) | 1.16 (0.81-1.66) |  | 125/469 (26.7%) | 1.36 (0.97-1.92) |  | 50/469 (10.7%) | 1.80 (1.23-2.64) |  |
| Stigma experienced in the community | No | 318/1801 (17.7%) | 1.00 | <0.001 |  |  |  |  |  |  |
|  | Yes | 244/541 (45.1%) | 3.97 (3.07-5.13) |  |  |  |  |  |  |  |
| Stigma experienced in a healthcare setting | No | 475/2158 (22.0%) | 1.00 | <0.001 | 386/2158 (17.9%) | 1.00 | <0.001 |  |  |  |
|  | Yes | 87/184 (47.3%) | 3.29 (2.16-5.02) |  | 155/184 (84.2%) | 24.39 (12.43-47.84) |  |  |  |  |

aOR: adjusted Odds Ratio ; CI: Confidence Interval ; n: Number of individuals experiencing the three types of stigma within groups; N: Total number of individuals within groups; P_w_: P value of the Wald test.

^a^: The aOR for sex is adjusted for age group; The aOR for age group is adjusted for sex; The aOR for all other predictor variables are adjusted for sex and age group.

^b^: A p value of less than 0.05 indicates that the predictor creates a statistically significant improvement in the fit of the model.
